# Genetic and environmental influences on educational disparities in adult weight change: an individual-based pooled analysis of 11 twin cohorts

**DOI:** 10.1038/s41366-026-02116-0

**Published:** 2026-06-12

**Authors:** Alvaro Obeso, Gabin Drouard, Aline Jelenkovic, Emanuela Medda, Corrado Fagnani, Virgilia Toccaceli, Antti Latvala, Sari Aaltonen, Sarah E. Medland, Scott D. Gordon, Jooyeon Lee, Soo Ji Lee, Joohon Sung, Hyojin Pyun, Glen E. Duncan, Dedra Buchwald, Juan R. Ordoñana, Juan F. Sánchez-Romera, Eduvigis Carrillo, Carol E. Franz, William S. Kremen, Robin P. Corley, Brooke M. Huibregtse, Patrik KE Magnusson, Ida K. Karlsson, Anna K. Dahl Aslan, Michael J. Lyons, Meike Bartels, Lannie Ligthart, Eco J. C. de Geus, Margaret Gatz, David A. Butler, René Pool, Anders Eriksson, Susanne Bruins, Nicholas G. Martin, Dorret I. Boomsma, Jaakko Kaprio, Karri Silventoinen

**Affiliations:** 1 Helsinki Institute for Demography and Population Health, University of Helsinki, Helsinki, Finland; 2 Department of Genetics, Physical Anthropology and Animal Physiology, Faculty of Science and Technology, University of the Basque Country (UPV/EHU), Bilbao, Spain; 3 Institute for Molecular Medicine Finland, HiLIFE, University of Helsinki, Helsinki, Finland; 4 Centre for Behavioural Sciences and Mental Health, Istituto Superiore di Sanità, Rome, Italy; 5 Institute of Criminology and Legal Policy, University of Helsinki, Helsinki, Finland; 6 QIMR Berghofer Medical Research Institute, Brisbane, Australia; 7 Institute of Health & Environmental, Seoul National University, Seoul, Korea; 8 Department of Epidemiology, Seoul National University School of Public Health, Seoul, Korea; 9 Washington State Twin Registry, Washington State University—Health Sciences Spokane, Spokane, WA USA; 10 Washington State Twin Registry, University of Washington, Seattle, WA USA; 11 Department of Human Anatomy and Psychobiology, University of Murcia, Murcia, Spain; 12 Murcia Institute for Biomedical Research (IMIB-Arrixaca), Murcia, Spain; 13 Department of Psychiatry, University of California San Diego, La Jolla, CA USA; 14 Institute for Behavioral Genetics, University of Colorado, Boulder, CO USA; 15 Department of Psychology and Neuroscience, University of Colorado, Boulder, CO USA; 16 Department of Medical Epidemiology and Biostatistics, Karolinska Institutet, Stockholm, Sweden; 17 School of Health Sciences, University of Skövde, Skövde, Sweden; 18 Department of Psychological and Brain Sciences, Boston University, Boston, MA USA; 19 Department of Biological Psychology, Vrije Universiteit Amsterdam, Amsterdam, the Netherlands; 20 Amsterdam Public Health Research Institute, Amsterdam, the Netherlands; 21 Center for Economic and Social Research, University of Southern California, Los Angeles, CA USA; 22 Health and Medicine Division, The National Academies of Sciences, Engineering and Medicine, Washington, DC USA; 23 cGEM, Institute of Genomics, University of Tartu, Tartu, Estonia; 24 Complex Trait Genetics, Center for Neurogenomics and Cognitive Research, Vrije Universiteit Amsterdam, Amsterdam, The Netherlands

**Keywords:** Epidemiology, Epidemiology

## Abstract

**Introduction:**

Educational attainment (EA) is negatively associated with body mass index (BMI), but less is known about the association between EA and adult BMI change. We analysed the role of genetic and environmental factors in the associations between EA and BMI trajectory components over adulthood.

**Data and methods:**

Pooled data from 59,490 twins aged 31–99 years (49% women) across 11 cohorts with EA and repeated measures of BMI were used. BMI trajectory components (baseline BMI and BMI change per decade) were estimated using linear mixed-effects (LME) and delta slope methods. EA was derived by regressing years of education on birth year and cohort. Associations between EA and BMI trajectories were evaluated with LME models in both cohort-specific and pooled data. Genetic and environmental contributions were evaluated using structural equation modelling.

**Results:**

EA was more strongly negatively associated with baseline BMI and BMI change (mean of 1.31 and 1.32 kg/m^2^ per decade in men and women, respectively) in women (*β* = –0.14 kg/m², 95% CI: –0.15 to –0.12; *β* = –0.02 kg/m²/decade, 95% CI: –0.03 to –0.01, respectively) than in men (*β* = –0.07, 95% CI: –0.08 to –0.06; *β* = –0.01, 95% CI: –0.02 to –0.001, respectively). The associations between baseline BMI and EA were explained by genetic factors in men (*r*_A_ = –0.10) and by both genetic (*r*_A_ = –0.17) and unique environmental factors (*r*_E_ = –0.07) in women. For BMI change, the associations with EA were explained by genetic factors (*r*_A_ = –0.04 in men; –0.06 in women).

**Conclusion:**

Individuals with higher EA tend to have lower baseline BMI and slower BMI increases across adulthood. These associations are primarily genetically mediated.

## Introduction

Educational attainment (EA) is a key social indicator in epidemiology [1], influencing health behaviours, occupational status, and lifetime earnings [2]. A large body of research has documented strong associations between EA and various health outcomes [3, 4] as well as mortality [5]. Obesity, typically measured using body mass index (BMI), is an important outcome linked to EA, as BMI strongly reflects fat mass and body fat percentage at the population level [6]. Studies across diverse populations have shown that higher personal EA is associated with lower adult BMI [7–10], and parental EA also influences offspring’s BMI in childhood and adulthood [11, 12]. Obesity prevalence has nearly tripled over the past 40 years [13, 14], potentially reinforcing educational health inequalities since BMI impacts multiple health outcomes [15–17] and mortality [16].

Both genetic and environmental factors influence EA and obesity. The development of obesity is affected by developmental, environmental, behavioural, and genetic factors, as well as their mutual interactions [18–20]. Twin studies have reported that from half to nearly 90% of individual differences in adult BMI are attributable to genetic differences [21–23]. This strong genetic influence is supported by genome-wide association studies (GWAS), which have identified numerous genetic variants associated with cross-sectional BMI in both childhood [24] and adulthood [25, 26]. In contrast, there is a paucity of genomic data specifically for longitudinal BMI. Although a few studies have examined this question, it remains unclear whether variants associated with cross-sectional BMI also influence longitudinal BMI trajectories. Previous studies have reported only modest genetic correlations (0.25–0.27) between polygenic risk scores derived from cross-sectional BMI GWAS and BMI change [27], suggesting that different sets of genetic factors contribute to cross-sectional BMI versus BMI change. Using a twin design, low heritability for BMI change across adulthood, remaining below 30% in both men and women at all stages, was found [28]. For EA, large-scale twin studies indicate that one-third to one-half of individual differences are genetically influenced [29]. GWAS have identified multiple single-nucleotide polymorphisms (SNPs) associated with EA, collectively explaining 3–16% of its variance [30, 31]. Furthermore, genetic correlations between EA and BMI have been reported in both twin (−0.20 in men and women) [32] and population-based studies (−0.20 to −0.25) [33].

Although the association between EA and BMI has been studied cross-sectionally, few studies have examined EA in relation to adult BMI change. Furthermore, the genetic and environmental contributions to these associations remain unclear. We aimed to address these gaps by analysing the phenotypic associations between EA and BMI trajectory components (baseline BMI and BMI change) across adulthood, as well as the underlying genetic and environmental influences on these associations, using longitudinal data from 11 twin cohorts across 8 countries.

## Data and methods

### Cohort

This study used data from the CODATwins project, combining twin cohorts worldwide with height and weight information, in collaboration with the BETTER4U consortium [34, 35]. Additional data on EA were available for some cohorts [36]. All participating cohorts obtained informed consent from participants in accordance with their respective local ethical board requirements. Thirteen cohorts with EA and longitudinal BMI data collected after age 18 were included: seven from Europe, four from the USA, and one each from Australia and South Korea, with two cohorts including only men. The cohorts were grouped into four regions: Europe, North America, East Asia, and Australia. BMI was calculated as weight divided by height squared (kg/m²) using self-reported (89%) or measured (11%) values. Education categories were converted to years by assigning the mean for each category and harmonised by regressing education years on birth year and cohort [36]. Residuals from this model represent individual deviations from expected education given birth year and cohort.

The initial database included 88,773 twins with EA and at least two BMI measures. Individuals reporting EA before age 30 were excluded to ensure completed education (*N* = 24,922). Baseline BMI and BMI change distributions were examined using scatter plots (Supplementary Figs. 1 and 2), and individuals with values exceeding 3 standard deviations from the mean were removed (*N* = 2400 for baseline BMI; *N* = 1961 for BMI change). The final sample comprised 59,490 twins (49% women) and 22,129 complete twin pairs (44% MZ, 47% same-sex DZ, 9% opposite-sex DZ) from 11 cohorts. The participant selection process is summarised in Supplementary Fig. 3.

### BMI trajectory components calculation

BMI trajectory components were derived using two methods. Participants with only two measurements had BMI change calculated via the delta method (difference between values divided by time elapsed), while those with three or more measurements used linear mixed-effects (LME) models, accounting for fixed and random effects and correlations between repeated observations [37]. Both approaches provided baseline BMI and the rate of BMI change per participant. LME models captured individual-level variation through random effects. To evaluate whether differences in BMI modelling contributed to heterogeneity, we conducted a random-effects meta-regression with the proportion of individuals with only two BMI measurements as a moderator (i.e. persons for whom BMI change was calculated by the delta method). The meta-regression showed that differences in the definition of BMI change were not significantly associated with baseline BMI (*p* = 0.294) or BMI change (*p* = 0.727), indicating that cohort heterogeneity was not explained by differences in BMI change definition. BMI change was annualised and scaled to per decade to allow comparison across participants with different follow-up times. Adult BMI change was assumed to be approximately linear between ages 18 and 60, so the annualised slope reflects each participant’s typical rate of BMI change.

### Association analysis

Associations between BMI trajectory components and EA were examined using LME models at both cohort-specific and pooled levels. All analyses were stratified by sex, given the known sex differences in BMI trajectories [23, 27, 38].

In cohort-specific analyses, baseline BMI was modelled as the dependent variable and EA as the independent variable, adjusting for baseline age and zygosity. BMI change was then modelled similarly, additionally including baseline BMI as a covariate. Heterogeneity across cohorts was assessed using Cochran’s *Q* test, which evaluates whether observed differences in effect estimates are compatible with chance under a common effect assumption [39]. Standard errors were derived from cohort-specific 95% confidence intervals (CI) assuming a standard multivariate normal distribution for BMI change and effect sizes.

When analysing the pooled data, a cohort-specific identifier was included as a categorical covariate to account for differences in baseline age, BMI, EA, and follow-up time across cohorts. We tested the linearity of EA associations with baseline BMI and BMI change by including quadratic age terms and applying flexible models using restricted cubic splines and generalised additive models (GAMs). In all LME models, random intercepts for family ID were included to account for the non-independence of observations due to familial relatedness.

Two sensitivity analyses were performed using pooled data. First, baseline smoking status (classified as current smokers, never smokers, and former smokers) was included as a covariate (*N* = 15,002) given its associations with EA [40–42] and BMI trajectories [43–45], treating smoking as a potential confounder. Participants with missing data on smoking, BMI trajectory components, or EA were excluded without imputation. Second, stratified analyses by life stages were conducted to examine age-specific associations, recognising that BMI trajectories vary across adulthood. For these analyses, we selected participants with the age at first and last BMI measurement in early midlife adulthood (30–50 years), midlife adulthood (51–65 years), and older adulthood (>66 years). To account for differences between cohorts, a cohort-specific identifier was included as a covariate in all models used for the sensitivity analyses. All statistical analyses were performed using R software (version 4.2.3) with the lme4 package (version 1.1-34). The Cochran’s Q tests were conducted using the metafor (version 3.8.1) and meta (version 6.3.0) R packages.

### Structural equation modelling

Data were analysed using structural equation modelling (SEM), leveraging differences in genetic relatedness between MZ twins, who are nearly genetically identical, and DZ twins, who share 50% of their segregating genes [46]. Trait variance was decomposed into additive genetic variance (A; correlation 1 in MZ, 0.5 in DZ), dominance genetic variance (D; correlation 1 in MZ, 0.25 in DZ), shared environmental variance (C; correlation 1 in both MZ and DZ), and unique environmental variance including measurement error (E; correlation 0 in both MZ and DZ). Because D and C cannot be estimated simultaneously in twins reared together, either an additive genetic/ shared environmental/unique environmental (ACE) or additive genetic/ dominance genetic/unique environmental (ADE) model was specified, with subsequent simplification to an AE model if appropriate. Consistency of intraclass correlations (ICCs) across cohorts justified pooling. Baseline models were first selected using ICCs calculated as the covariance between twins divided by total trait variance, with 95% CIs obtained from the F distribution [47]. The best-fitting model was then compared to the more parsimonious AE model and further tested against submodels excluding (i) sex differences and (ii) sex-specific genetic effects using the *χ*²-distributed –2 log-likelihood test.

A bivariate Cholesky decomposition was conducted to examine genetic and environmental contributions to the associations between EA and BMI trajectory components, both at the cohort-specific level and in pooled data to maximise power. This model-free approach decomposes variance and covariance between two traits into additive genetic (A), shared environmental (C), and unique environmental (E) components via independent latent factors [48]. Standardised genetic and environmental correlations were calculated by dividing each covariance by the square root of the product of the corresponding trait variances, yielding genetic (*r*_A_), shared environmental (*r*_C_), and unique environmental (*r*_E_) correlations with 95% CIs estimated by maximum likelihood [49]. Heterogeneity across cohorts was assessed using Cochran’s *Q* test [39]. Sex differences were modelled with separate SEM subsets for male-male (MZM, DZM) and female-female (MZF, DZF) twin pairs, while opposite-sex DZ (OSDZ) pairs were included with freely estimated genetic correlations. Analyses were conducted in R (version 4.2.3) using OpenMx (version 2.21.11), and Cochran’s *Q* tests were performed using the metafor (version 3.8.1) and meta (version 6.3.0) packages.

## Results

Descriptive characteristics of the cohorts included in the study after applying exclusion criteria are shown in Table 1. The Vietnam Era Twin Study of Aging (VETSA) had the highest baseline BMI (29.07 kg/m²) and the oldest baseline age (56.29 years), whereas the Netherlands Twin Registry had the lowest baseline BMI (21.62 kg/m²) and the youngest baseline age (31.26 years). The greatest BMI change per decade was observed in the Washington State Twin Registry (1.40 kg/m²), while the lowest was seen in the Colorado Twin Registry and Korea Twin-Family Register (KT-FR) (0.60 kg/m²). EA was highest in the Washington State Twin Registry (15.26 years) and lowest in the Finnish Old Cohort (8.05 years). The mean follow-up time was 15.83 years, with the NAS-NCR Twin register having the longest follow- up (22.60 years) and the Italian Twin Registry the shortest follow-up time (4.23 years). Cohort-specific associations between baseline BMI and BMI change were significant in most cohorts for both sexes, except in VETSA, KT-FR, and NAS-NRC Twin Registry for men, and KT-FR and Swedish Twin Registry for women. Estimates ranged from 0.03 to 0.32 in men and 0.04 to 0.57 in women (Supplementary Fig. 4). Cohort-specific and pooled data information on BMI trajectories are shown in Supplementary Table 1 and Supplementary Figs. 5 and 6 for men and women, respectively.

**Table 1:** Descriptive information of the cohorts included in the database after the application of exclusion criteria.

Geographic area and cohorts	General information			Follow up information			Baseline information			Longitudinal information		Information on education	
	Age range	*N* of individuals	% women	Maximum N of measures	Mean follow-up time (years)	% Individuals with 2 BMI measures	Mean baseline age (years)	Mean Baseline BMI	Mean birth year	BMI change per ten years		Mean education years	
										Mean	SD	Mean	SD
*Europe*													
Finnish old cohort	31–100	20,939	53%	4	19.21	42%	33.65	23.10	1951	0.80	0.40	8.05	3.29
Italian Twin Registry	31–78	1280	67%	4	4.23	82%	34.19	22.27	1973	0.80	3.40	13.40	2.26
Murcia Twin Register	31–89	1298	64%	8	7.39	85%	35.97	25.84	1963	0.80	0.70	10.67	3.91
Netherlands Twin Register	31–91	3961	66%	13	10.65	21%	31.26	21.62	1968	0.90	0.40	13.96	2.45
Swedish Twin Register	31–99	8255	46%	4	19.90	80%	39.77	24.51	1940	1.00	0.90	11.96	3.08
*North America*													
Colorado Twin Registry	31–34	1478	56%	3	6.62	94%	31.69	22.68	1985	0.60	0.40	14.51	2.17
NAS-NRC Twin Registry	31–82	8692	0%	4	22.60	49%	44.26	22.22	1923	0.80	0.60	13.52	3.09
Vietnam Era Twin Study of Aging	51–66	743	0%	2	5.76	100%	56.29	29.07	1950	0.90	3.70	13.88	2.09
Washington State Twin Registry	31–97	5877	66%	6	6.67	56%	35.67	24.81	1970	1.40	5.70	15.26	2.38
*Asia*													
Korea Twin-Family Register	31–79	200	65%	4	4.37	56%	38.73	23.07	1968	0.60	3.00	14.24	3.92
*Australia*													
Queensland Twin Register	31–92	6767	51%	9	14.00	42%	33.18	22.75	1959	1.10	4.20	15.16	2.07
*World*													
All cohorts	31–100	59,490	49%	2-13	15.83	52%	37.89	22.54	1952	0.90	2.60	11.81	4.15

Descriptive information of the 13 cohorts that form the CODATwins database used in the current study after application of exclusion criteria.

*N* number, *SD* standard deviation, *NAS-NRC* National Academy of Sciences-National Research Council.

**Fig. 1:**
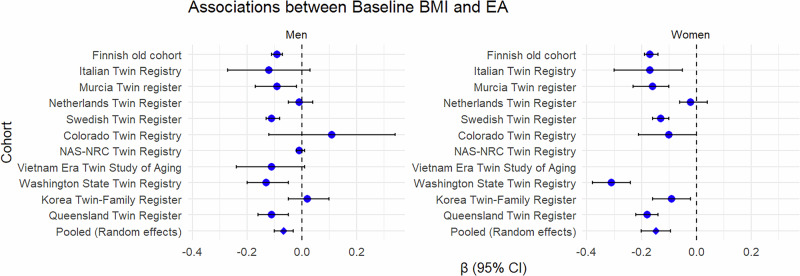
Association between educational attainment and baseline BMI by sex and twin cohort. Forest plots showing the association between educational attainment and baseline BMI in men (left) and women (right) across multiple twin cohorts. Each dot represents the point estimate of the association for a specific cohort, with horizontal lines indicating 95% confidence intervals. Cochran’s *Q* test indicates significant heterogeneity across cohorts (Men: *Q* = 120.97, *p* = 3.95e-20; Women: *Q* = 125.38, *p* = 4.06e-22). BMI body mass index.

**Fig. 2:**
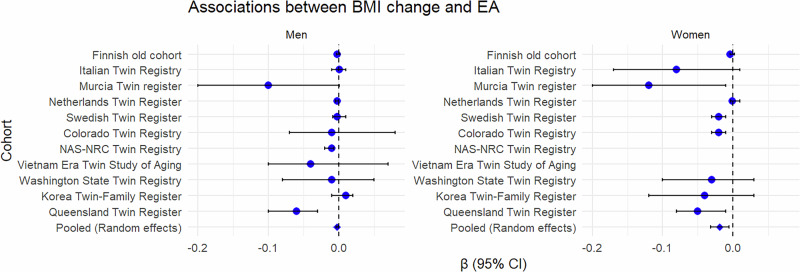
Association between educational attainment and BMI change by sex and twin cohort. Forest plots showing the association between educational attainment and BMI change in men (left) and women (right) across multiple twin cohorts. Each dot represents the point estimate of the association for a specific cohort, with horizontal lines indicating 95% confidence intervals. Cochran’s *Q* test indicates significant heterogeneity across cohorts (Men: *Q* = 42.88, *p* = 2.35e-05; Women: *Q* = 65.00, *p* = 4.04e-10). BMI body mass index.

Figure 1 shows the cohort-specific associations between EA and baseline BMI, while Fig. 2 presents the results for BMI change over a decade (the exact estimates are available in Supplementary Table 2). Higher EA was generally linked to lower baseline BMI and smaller BMI increases. Statistically significant associations for baseline BMI were observed in six cohorts of men and eight cohorts of women, and for BMI change in two cohorts of men and four cohorts of women. Cochran’s *Q* tests indicated significant heterogeneity across cohorts for all outcomes in both sexes (all *p* < 0.001).

The results of the associations of EA with baseline BMI and BMI change using pooled data (N = 59,490) are displayed in Table 2. EA was negatively associated with baseline BMI and BMI change in both men and women. In men, a one standard deviation (SD) higher EA (approximately 3 years) was associated with a 0.07 kg/m² lower baseline BMI and a 0.01 kg/m² per decade slower increase in BMI. In women, a one SD higher EA (2.80 years) was associated with a 0.14 kg/m² lower baseline BMI and a 0.02 kg/m² per decade slower rate of BMI change. Linearity tests confirmed the robustness of associations.

**Table 2:** Association study of EA with baseline BMI (kg/m^2^) and BMI change (kg/m^2^ per 10 years) in pooled data, by sex.

Variables of study	Men (*N* = 33,763)			Women (*N* = 31,314)		
	β	95% CI		β	95% CI	
		LL	UL		LL	UL
Baseline BMI	−0.07	−0.08	−0.06	−0.14	−0.15	−0.12
BMI change	−0.01	−0.02	–1.26e-03	−0.02	−0.03	−0.01

Associations analyses were carried out using LME models. The models are: (i) baseline BMI ~ EA + cohort ID + Zygosity + Baseline Age + (1|Family ID) and ii) BMI change~ EA + cohort ID + Zygosity + Baseline Age + baseline BMI + (1|Family ID). Variables were harmonised prior to analysis, and results are interpreted as effect sizes per 1 SD change in EA. The estimates are displayed along with the 95% confidence interval.

*BMI* body mass index, *CI* confidence interval, *LL* lower limit, *UL* upper limit.

LME models detected a significant quadratic age effect on BMI slope (*p* < 0.001) but no significant EA–age interactions (men: *p* > 0.05; women: *p* = 0.167 and 0.648). GAMs showed non-linear age effects (men: edf = 3.47, *F* = 38.91, *p* < 0.001; women: edf = 4.72, *F* = 27.15, *p* < 0.001) without significant EA–age interactions (men: *p* = 0.343; women: *p* = 0.277). Restricted cubic spline models yielded consistent EA effects, with only minor late-age interactions that did not improve model fit (men: *χ*² = 6.34, df = 4, *p* = 0.175; women: χ² = 7.53, df = 4, *p* = 0.111). The sensitivity analyses results—including (i) adjustment for smoking and (ii) stratification of adulthood into stages—are shown in Supplementary Tables 3 and 4. Associations remained largely significant after adjusting for smoking (N = 15,002) (Supplementary Table 3). Age-stratified analyses (*N* = 19,451; 2892; 545 individuals in early midlife adulthood, midlife adulthood, and older adulthood, respectively) largely confirmed the primary findings but revealed some sex- and age-specific variation (Supplementary Table 4). In men, EA was negatively associated with baseline BMI only in early midlife adulthood (*β* = −0.09) and was not associated with BMI change at any stage. In women, EA was significantly associated with baseline BMI across all stages (*β* = −0.12 to −0.15) and with BMI change until older adulthood (*β* = −0.02 to −0.10).

We began genetic modelling by selecting the best fitting model based on MZ and DZ ICCs (Supplementary Table 5). The ACE model was selected as the baseline model because MZ correlations were less than twice DZ correlations. Removing the shared environment variance did not impact the model fit for baseline BMI or BMI change, but it did affect EA (Supplementary Table 6). No sex-specific genetic effects were detected. Therefore, we used the AE model for BMI and an ACE model for EA in the subsequent analyses, both without qualitative sex differences but including sex-specific variance components.

The results of the genetic and environmental factors influencing the variation of baseline BMI, BMI change, and EA are displayed in Table 3. For baseline BMI, the genetic additive effect explained most of the variation in men (*a*^2^ = 0.81) and women (*a*^2^ = 0.79). The variation in BMI change was mainly explained by unique environmental factors in men (*e*^2^ = 0.79) and women (*e*^2^ = 0.81). For EA, additive genetic effects explained a substantial part of the variation in men (*a*^2^ = 0.41) followed by shared environmental effects (*c*^2^ = 0.30), while in women, shared environmental effects explained most of the variation (*c*^2^ = 0.42), followed by additive genetic effects (*a*^2^ = 0.31).

**Table 3:** Relative proportions of the variability of baseline BMI, BMI change and EA explained by additive genetic, shared environmental and unique environmental variance components with 95% confidence intervals.

Variables of study	Additive genetic effect			Shared environmental effect			Unique environmental effect		
	*a*^2^	95% CI		*c*^2^	95% CI		*e*^2^	95% CI	
		LL	UL		LL	UL		LL	UL
Men									
Baseline BMI	0.81	0.79	0.82	-	-	-	0.19	0.18	0.20
BMI change	0.21	0.17	0.23	-	-	-	0.79	0.76	0.82
EA	0.41	0.37	0.45	0.30	0.26	0.33	0.29	0.27	0.30
Women									
Baseline BMI	0.79	0.78	0.80	-	-	-	0.21	0.20	0.22
BMI change	0.19	0.16	0.21	-	-	-	0.81	0.78	0.83
EA	0.31	0.27	0.35	0.42	0.38	0.44	0.27	0.26	0.28

Full AE model was selected for baseline BMI and BMI change, while full ACE model for EA.

*a*^2^ proportion of total variance explained by additive genetic effects, *c*^2^ proportion of total variance explained by shared environmental effects, *e*^2^ proportion of total variance explained by unique environmental effects, *LL* Lower limit, *UL* upper limit.

**Fig. 3:**
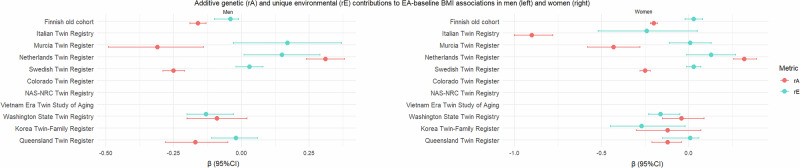
Cohort specific additive genetic and unique environmental correlations underpinning the associations between baseline BMI and EA in men and women. Forest plot showing estimates of genetic (*r*_A_, red) and unique environmental (*r*_E_, teal) correlations obtained from the associations between BMI baseline BMI and EA across multiple cohorts in men in the left and women in the right. Points represent correlation estimates, and horizontal lines represent 95% confidence intervals. Cochran’s *Q* tests indicate significant heterogeneity for baseline BMI (*r*_A_: *Q* = 195.14, *p* = 2.05e-39; *r*_E_: *Q* = 21.27, *p* = 3.38e-03) and BMI change (*r*_A_: *Q* = 1.76, *p* = 0.62; *r*_E_: *Q* = 14.72, *p* = 2.06e-03). BMI body mass index, CI confidence interval, r_A_ additive genetic correlations, r_E_ unique environmental correlations.

**Fig. 4:**
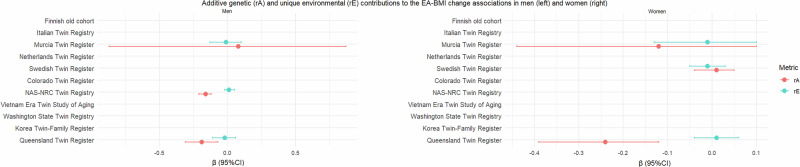
Cohort specific additive genetic and unique environmental correlations between BMI change and EA in men and women. Forest plot depicting cohort-level estimates of additive genetic (*r*_A_, red) and unique environmental (*r*_E_, teal) correlations between BMI change and EA in men in the left and women in the right. Each point represents a correlation estimate, with horizontal lines indicating 95% confidence intervals. Cochran’s *Q* tests indicate significant heterogeneity for baseline BMI (*r*_A_: *Q* = 441.93, *p* = 1.52e-89; *r*_E_: *Q* = 46.10, *p* = 1.37e-06) and BMI change (*r*_A_: *Q* = 0.49, *p* = 0.92; *r*_E_: *Q* = 12.59, *p* = 5.58e-03). BMI body mass index, CI confidence interval, r_A_ additive genetic correlations, r_E_ unique environmental correlations.

Associations between BMI trajectory components and EA were primarily explained by genetic and unique environmental factors (Table 4). In men, the association between EA and baseline BMI was largely influenced by additive genetic factors (*r*_A_ = –0.10), whereas in women, both additive genetic (*r*_A_ = –0.17) and unique environmental factors (*r*_E_ = –0.07) contributed to the association. For BMI change per decade, associations with EA were modestly influenced by additive genetic factors in men (*r*_A_ = –0.04) and women (*r*_A_ = –0.06). Cohort-specific bivariate Cholesky results are shown in Figs. 3 and 4 (exact estimates in Supplementary Table 7), revealing variation in genetic and environmental contributions across cohorts. Cochran’s Q tests indicated significant heterogeneity for *r*_A_ and *r*_E_ in baseline BMI in both sexes (all *p* < 0.001). For BMI change, heterogeneity was observed for *r*_E_ in men (*p* < 0.001) and *r*_A_ in women (*p* < 0.001).

**Table 4:** Contribution of additive genetic and unique environmental factors to the previously identified associations between BMI trajectory components (baseline BMI and BMI change) and EA by sex.

Variables of study	Men						Women					
	Additive genetic correlation			Unique environmental correlations			Additive genetic correlation			Unique environmental correlation		
	*r*_A_	95% CI		*r*_E_	95% CI		*r*_A_	95% CI		*r*_E_	95% CI	
		LL	UL		LL	UL						
Baseline BMI	−0.10	−0.12	−0.08	−0.01	−0.04	0.01	−0.17	−0.19	−0.15	−0.07	−0.09	−0.05
BMI change	−0.04	−0.07	−0.01	−0.01	−0.03	0.01	−0.06	−0.10	−0.02	−0.01	−0.03	0.01

Additive genetic (*r*_A_) and unique environmental (*r*_E_) correlations of EA with baseline BMI and BMI change were obtained from structural equation models, which decompose phenotypic covariation into genetic and environmental effects.

*r*_A_ Additive genetic correlation coefficient, *r*_E_ Specific environmental correlation coefficient, *LL* Lower limit, *UL* Upper limit.

## Discussion

Our findings showed that EA was negatively associated with baseline BMI and BMI change in both men and women. Moreover, genetic and environmental factors explained the variation in these traits and their associations. Individuals with higher education tended to have lower baseline BMI and slower BMI increases across adulthood. However, the associations varied by age and sex when stratified by life stage: men showed significant associations only for baseline BMI in early midlife adulthood (30–50 years), whereas women had significant associations with baseline BMI across all stages (strongest in older adulthood) and with BMI change up to older adulthood (strongest in midlife adulthood). This pattern may reflect reduced statistical power in stratified analyses but may also indicate that the protective effect of education on BMI trajectories differs across adulthood, particularly among women, and is most pronounced during the middle and older stages.

These results align with previous studies showing a higher risk of obesity among individuals with lower EA [50–52]. Longitudinal research has further found that those with higher EA tend to gain less weight over time and are less likely to transition into overweight or obesity categories during adulthood [53, 54]. EA may also moderate the impact of socioeconomic disadvantages on obesity, potentially buffering against weight gain in low-income groups by promoting protective health behaviours [55]. Differences in health literacy, dietary patterns, and physical activity associated with EA have been suggested as key mechanisms underlying this relationship [56, 57]. However, to our knowledge, no prior research has specifically examined the contributions of genetic and environmental factors to the association between EA and BMI change over time.

Cohort-specific analyses first indicated that associations between baseline BMI and BMI change were generally positive, consistent with previous findings from the same CODATwins cohorts, suggesting that higher initial BMI modestly predicts later BMI trajectories in both men and women [28]. At the same time, substantial heterogeneity remained in the associations with EA, although meta-regression analysis indicated that this was not explained by methodological differences in BMI change definitions across cohorts. Thus, heterogeneity likely reflects differences in sample size, age distribution, and follow-up duration. Larger cohorts showed consistent negative associations between baseline BMI and EA, whereas smaller cohorts displayed wider CIs and less consistent patterns, particularly for BMI change. Temporal and cultural factors likely contribute to these differences, as variations in public health policies, nutritional environments, and socioeconomic conditions can modulate how education translates into health behaviours and BMI outcomes, even though identifying features of an obesogenic environment remains challenging [58]. For example, earlier-born cohorts may have experienced different educational opportunities and dietary environments than younger cohorts. Similarly, European cohorts with longer follow-up may capture more gradual BMI trajectories, whereas smaller or shorter follow-up cohorts may have less power to detect change. Differences in data collection timing further complicate comparisons, making it difficult to disentangle age, period, and country-level effects.

Genetic twin modelling revealed distinct sources of individual variance for the traits examined. For baseline BMI, additive genetic effects were the primary contributors to differences in both men and women, with heritability estimates (~0.80) similar to those reported in early adulthood using CODATwin cross-sectional data [20]. In contrast, variability in BMI change was predominantly explained by unique environmental factors, reflecting the influence of individual-specific experiences or exposures, including weight gain after pregnancy in women. These results are in line with prior studies of CODATwin database with larger cohort samples [28]. For EA, sources of variance differed by sex: in men, additive genetic effects dominated, followed by shared environmental influences slightly exceeding unique environmental effects. In women, shared environmental factors contributed most strongly, followed by additive genetic effects, consistent with previous findings [29].

Our findings highlight the underlying factors linking EA and BMI trajectories across adulthood. In men, the associations of EA with both baseline BMI and BMI change were primarily influenced by additive genetic factors, suggesting that some of the genetic factors related to higher education also contribute to lower BMI levels and slower BMI increase. However, the magnitude of the genetic correlations was modest, indicating that the shared genetic contribution is limited. In women, the association between baseline BMI and EA was shaped by both additive genetic and unique environmental factors, whereas the association with BMI change was primarily explained by additive genetic factors. This sex difference may indicate stronger genetic links between EA and BMI in men. Historically, restricted access to higher education may have limited women’s ability to realise their genetic potential for EA or made their educational trajectories more dependent on non-systemic factors, weakening the EA–BMI genetic correlation and increasing environmental variation. In addition, BMI in women may be more sensitive to reproductive factors and sociocultural pressures [59–62], further increasing the contribution of unique environmental influences. It should also be noted that potential gene–environment interactions were not explicitly modelled in the present analyses; depending on whether environmental exposures are shared or individual-specific, such interactions may be partly captured within the additive genetic or unique environmental components in the twin design. Therefore, although higher education is associated with healthier BMI trajectories, the modest genetic correlations and the possibility of unmodeled processes such as gene–environment interplay limit causal interpretation. Moreover, cohort-specific analyses indicated variation in genetic and environmental contributions across populations, reflecting differences in social, cultural, healthcare, and age contexts. While genetic influences were relatively consistent, unique environmental effects varied more across cohorts, suggesting that the interplay between genetics, education, and BMI trajectories is context dependent. Accordingly, the protective association of education with BMI may differ depending on environmental exposures and cohort-specific characteristics.

Our findings highlight the importance of considering sex and cohort-specific contexts when interpreting socioeconomic effects on long-term health. Previous work on genetic and environmental contributions to education-related BMI differences found that the negative association between baseline BMI and EA was largely driven by additive genetic effects, with only modest positive unshared environmental effects in women. That study also reported that the same genetic factors influencing baseline BMI and EA contributed to the EA–weight change association [32]. However, no prior research has examined the genetic and environmental bases of EA associations with BMI trajectory components. Our study extends this literature by using a longitudinal, trajectory-based approach, allowing decomposition of genetic and environmental contributions not only to baseline BMI but also to BMI change patterns over adulthood.

The current study has several strengths, most notably the inclusion of a large sample of twins from multiple cohorts with EA and longitudinal BMI data. Information on smoking status allowed us to account for this important confounder, enhancing the validity of observed associations between BMI trajectory components and EA. This comprehensive dataset strengthens our ability to disentangle genetic and environmental contributions over time and across individuals. However, there are limitations. First, the absence of data from low- and middle-income countries restricts generalisability. Second, our analyses include only completed levels of education and thus, incorporating additional factors such as academic performance, cognitive ability, motivation, and other influences on educational outcomes could provide deeper insight into how BMI and its changes relate to EA and the genetic and environmental mechanisms underlying these associations. Assortative mating, choosing partners based on similarity in traits, can affect EA, a highly heritable trait [29], by increasing the genetic correlation between spouses. This may elevate the additive genetic correlation of DZ twins and full siblings above 0.5, potentially inflating shared environmental estimates and biasing separation of genetic and environmental contributions in classical twin designs [63, 64]. In this study, accounting for assortative mating was not possible due to missing spouse information in several cohorts. Finally, while smoking data were available, information on other potential modifiers, such as nutrition, physical activity, and parity, was lacking. Parity history may influence BMI trajectories in women, and historical fertility patterns could contribute to sex-specific differences. It is also possible that genetic factors can contribute to the association between parity and education [65] and thus it is possible that parity can also contribute to the genetic correlation between education and weight gain. Further studies having longitudinal information on parity and other factors affecting weight change would be important to identify mechanisms behind the observed associations.

In conclusion, associations between EA and adult BMI trajectories are primarily shaped by additive genetic factors, with minimal influence from unique environmental factors. EA is negatively associated with baseline BMI and BMI change, with genetic factors largely explaining these associations in men and both genetic and unique environmental factors contributing in women, especially for baseline BMI. These results underscore the importance of dynamic weight measures and reveal sex-specific differences in BMI aetiology and its relationship with education. While the results are based on a large twin sample from multiple cohorts, which enhances robustness, generalisability may be somewhat limited to populations with similar socio-cultural and educational contexts.

## Supplementary information

Supplementary material

## Data Availability

The data used in this study is owned by third parties (the individual twin cohorts) and made available to this study in condition that they will be used only in this meta-analysis. The data can be used based on the same principles as used in this study (more information from Karri Silventoinen karri.silventoinen@helsinki.fi).

## Code availability

All R scripts are available from the corresponding author.
