# Supplementary material for: Genetic and environmental influences on educational disparities in adult weight change: an individual-based pooled analysis of 11 twin cohorts
Source: Int J Obes (Lond). 2026 Jun 12;50(8):1828–37. doi: 10.1038/s41366-026-02116-0 (PMC13461365; doi:10.1038/s41366-026-02116-0)
Supplement: Supplementary file 1 [file 41366_2026_2116_MOESM1_ESM.pdf]

Supplementary Figure 1. Baseline BMI distribution for identifying outliers.

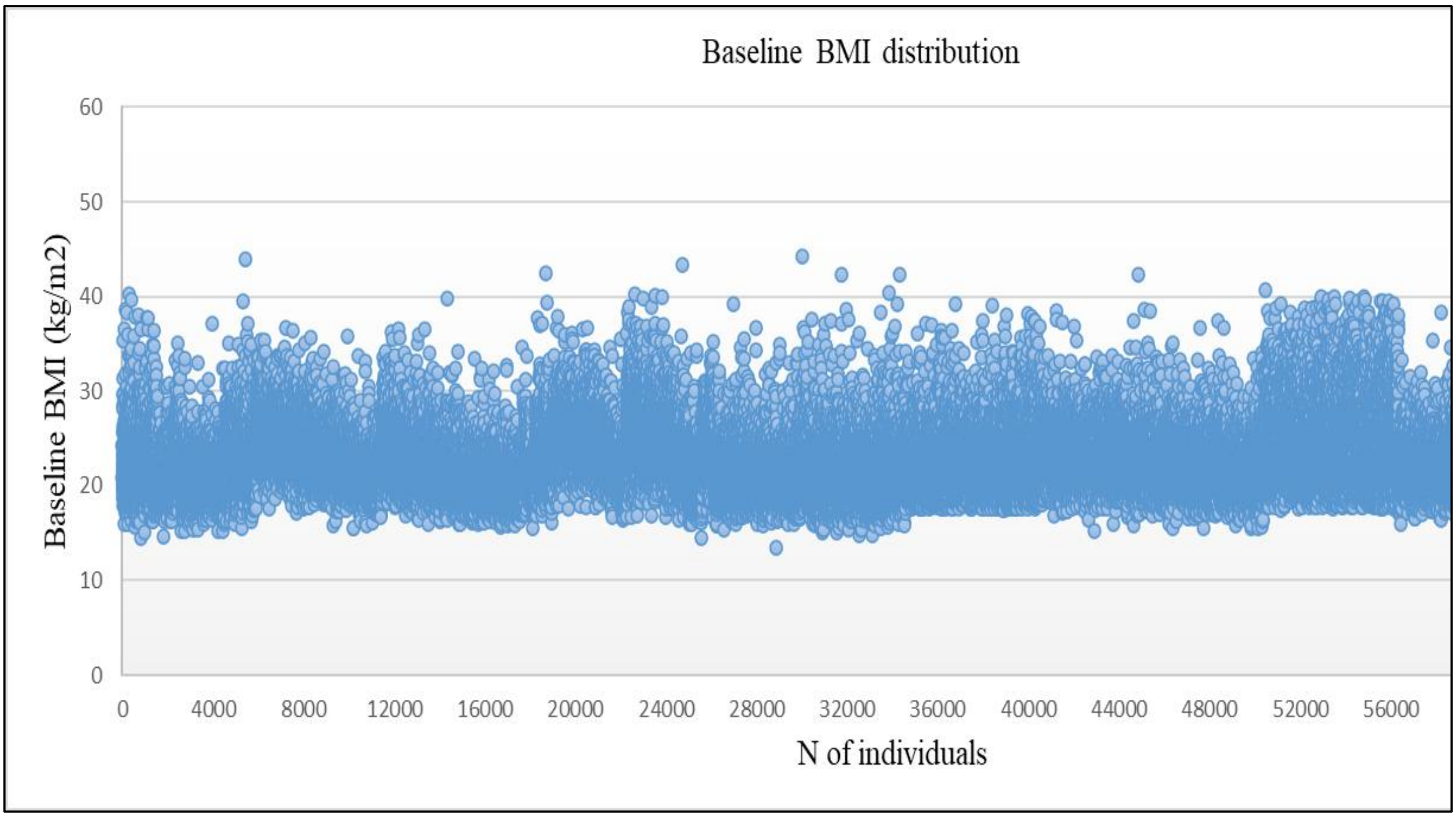

Caption: Scatter plot displaying baseline BMI values is shown in the figure. Individuals with BMI values greater than 3 standard deviations from the mean were considered outliers and removed prior to analysis. **Abbreviations:** BMI: body mass index.

Supplementary Figure 2. BMI changes distribution for identifying outliers.

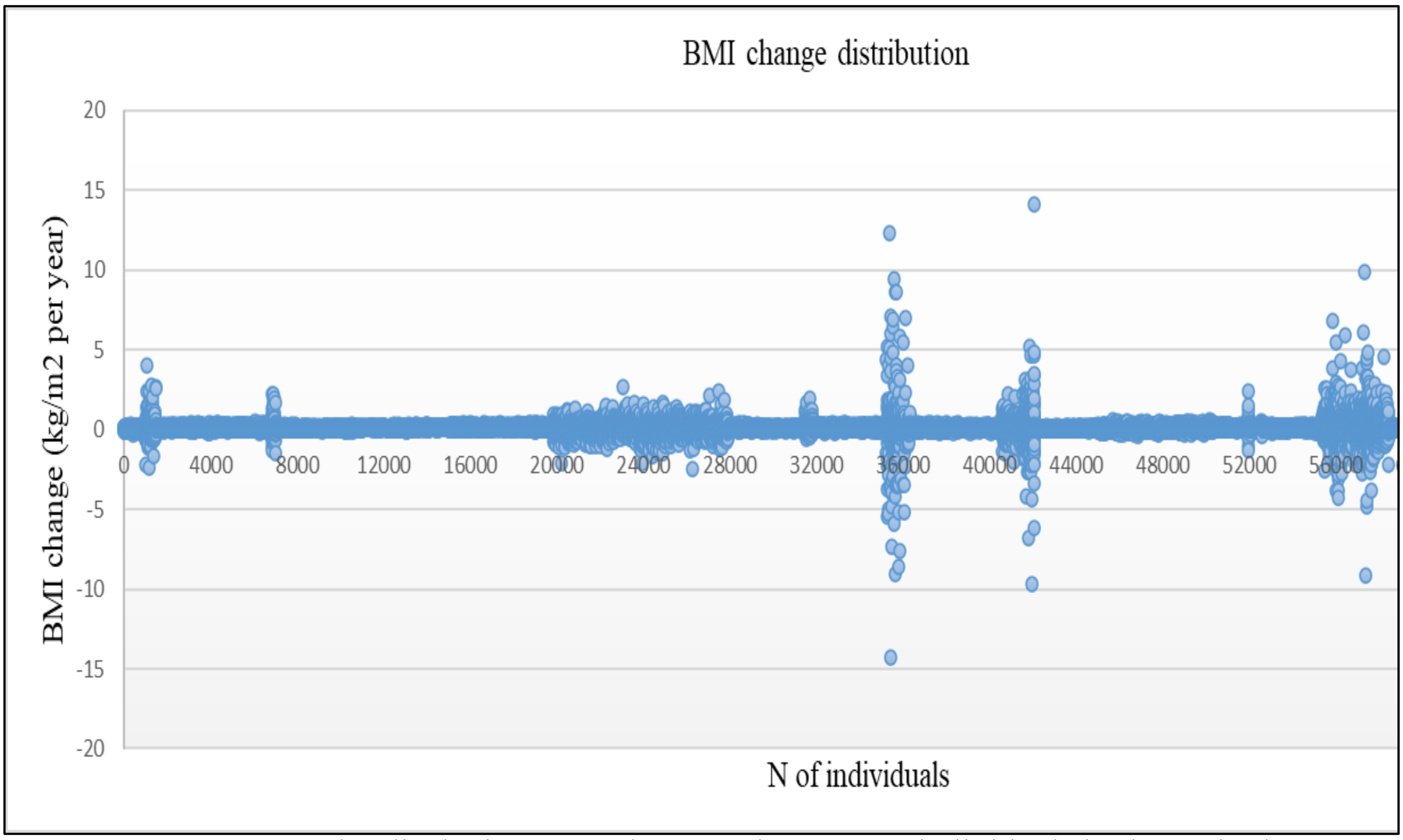

Caption: Scatter plot displaying BMI change values across individuals is shown in the figure. Individuals with BMI change values greater than 3 standard deviations from the mean were considered outliers and removed prior to analysis. **Abbreviations:** BMI: body mass index

Supplementary Figure 3: Flow chart illustrating the selection and inclusion process of participants in the current study.

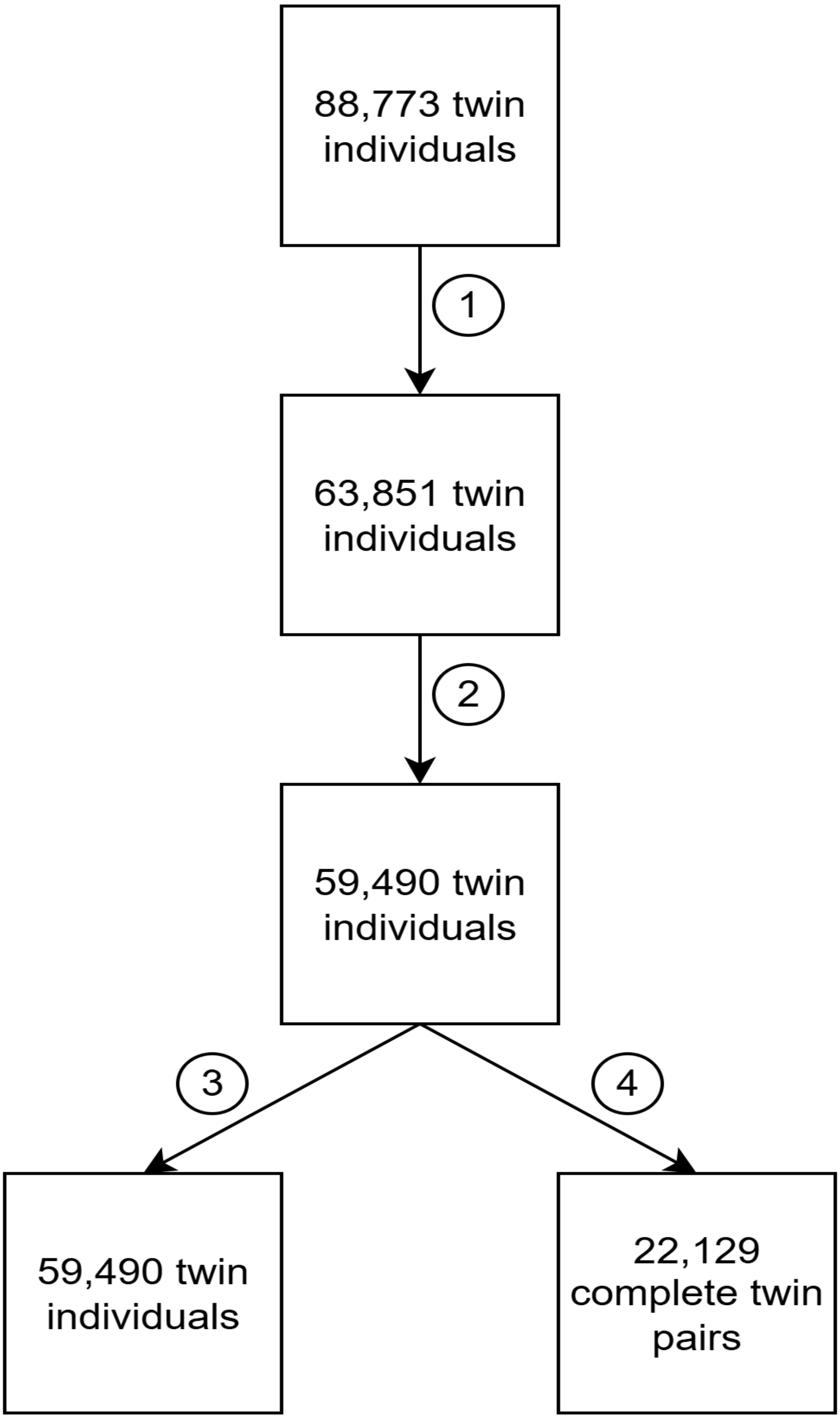

Caption: The study began with 88,730 individuals drawn from cohorts that included information on educational attainment and repeated BMI measurements. (1) Participants with educational data reported only before age 30 were excluded. (2) We then examined the distribution of baseline BMI and BMI change, excluding those with values exceeding three standard deviations from the mean. (3) Associations at the individual level were analyzed

using linear mixed-effects (LME) models. (4) Finally, structural equation modelling—both univariate and bivariate—was conducted using complete twin pairs.

Supplementary Figure 4. Cohort-specific associations between baseline BMI and BMI change by sex

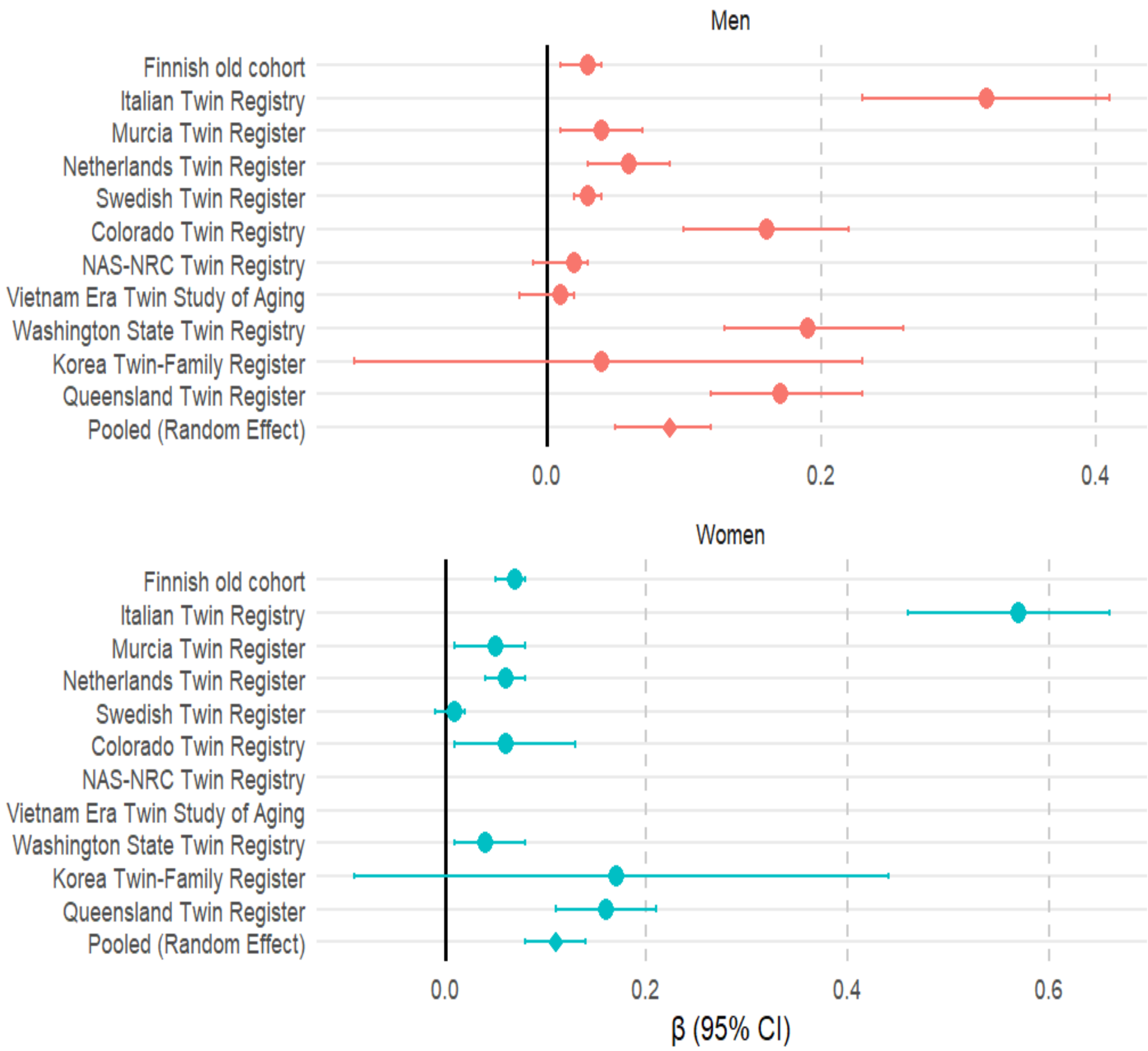

Caption: Forest plot showing cohort-specific associations between baseline BMI and BMI change in men (top panel) and women (bottom panel), with pooled random-effects estimates included. **Abbreviations:** BMI: body mass index; CI: confidence interval; NAS-NRC: National Academy of Sciences-National Research Council.

Supplementary Figure 5: Cohort-specific and pooled data (brown) BMI trajectories in men.

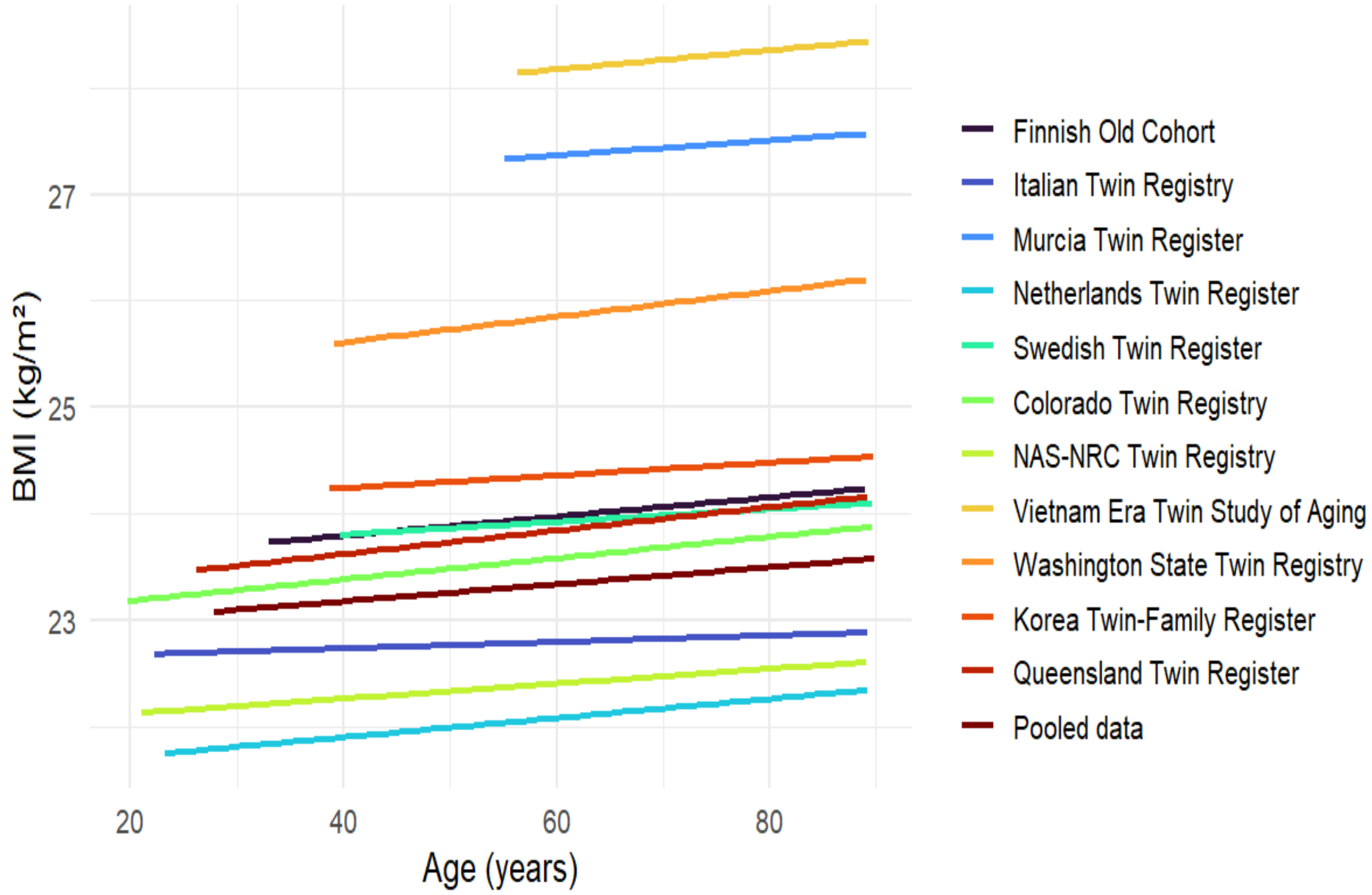

Caption: Line plot showing BMI trajectories in men for both cohort-specific data and pooled data (brown). Trajectories are constructed based on mean baseline age, mean baseline BMI, and mean BMI change rate**. Abbreviations:** BMI: body mass index; NAS-NRC: National Academy of Sciences-National Research Council.

Supplementary Figure 6. Cohort-specific and pooled data (brown) BMI trajectories in women.

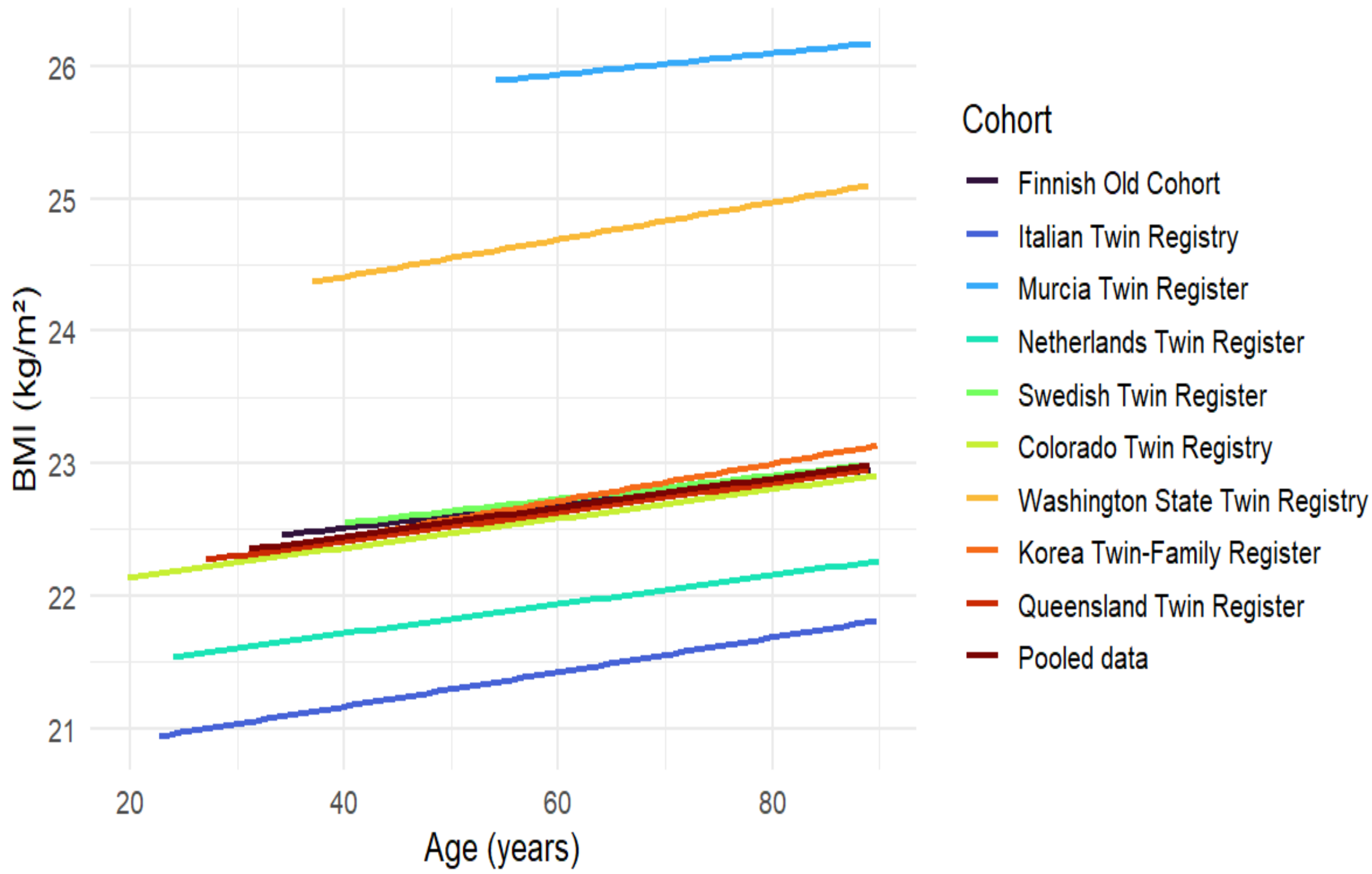

Caption: Line plot showing BMI trajectories in men for both cohort-specific data and pooled data (brown). Trajectories are constructed based on mean baseline age, mean baseline BMI, and mean BMI change rate**. Abbreviations:** BMI: body mass index.

Supplementary Table 1. Cohort-specific and pooled data on BMI trajectory components and baseline age by sex.

	**Men**			**Women**		
	**Mean baseline age (SD)**	**Mean baseline BMI (SD)**	**Mean BMI change (SD)**	**Mean baseline age (SD)**	**Mean baseline BMI (SD)**	**Mean BMI change (SD)**
Europe						
Finnish old cohort	33.02 (11.93)	23.73 (2.98)	0.09 (0.17)	34.22 (13.62)	22.46 (3.44)	0.09 (0.20)
Italian Twin Registry	22.22 (6.90)	22.68 (3.01)	0.03 (0.52)	22.72 (7.41)	20.94 (3.05)	0.13 (0.77)
Murcia Twin Register	55.08 (7.91	27.33 (3.94)	0.07 (0.19)	54.19 (7.98)	25.89 (4.63)	0.08 (0.23)
Netherlands Twin Register	23.24 (10.23)	21.75 (2.68)	0.09 (0.12)	23.99 (10.16)	21.54 (2.93)	0.11 (0.13)
Swedish Twin Register	39.63 (4.91)	23.80 (2.45)	0.06 (0.07)	40.10 (5.12)	22.55 (2.93)	0.09 (0.09)
North America						
Colorado Twin Registry	19.69 (1.68)	23.18 (3.45)	0.10 (0.26)	19.73 (1.67)	22.14 (3.73)	0.11 (0.35)
NAS-NRC Twin Registry	21.09 (5.67)	22.13 (2.49)	0.07 (0.05)	-	-	-
Vietnam Era Twin Study of Aging	56.29 (2.58)	28.14 (3.19)	0.09 (0.19)	-	-	-
Washington State Twin Registry	39.11 (18.57)	25.60 (4.03)	0.12 (0.54)	37.02 (17.11)	24.37 (4.44)	0.14 (0.54)
Asia						
Korea Twin-Family Register	38.70 (7.21)	24.23 (2.46)	0.06 (0.21)	38.75 (7.42)	22.42 (2.91)	0.14 (0.43)
Australia						
Queensland Twin Register	26.22 (8.37)	23.47 (2.99)	0.11 (0.39)	27.03 (9.39)	22.27 (3.24)	0.11 (0.44)
World						
Pooled data	27.88 (11.77)	23.08 (3.04)	0.08 (0.23)	31.10 (13.09)	22.35 (3.52)	0.11 (0.35)

Caption: Mean baseline age, baseline BMI, and annual BMI change along with the standard deviation are shown for men and women across each of the included twin cohorts and at pooled data level. **Abbreviations:** BMI: body mass index; SD: Standard deviation; NAS-NRC: National Academy of Sciences-National Research Council.

Supplementary Table 2. Associations of EA with baseline BMI (kg/m^2) and BMI change (kg/m^2 per ten years) in each of the cohort included in the study, by sex.

	Men			Women		
	β	95%CI		β	95%CI	
		LL	UL		LL	UL
Europe						
Finnish old cohort	N= 9,879			N= 11,059		
Baseline BMI	-0.09	-0.11	-0.07	-0.17	-0.19	-0.14
BMI change	-1.72e-03	-2.71e-03	1.69e-03	-3.92e-03	-4.05e-03	1.80e-03
Italian Twin Registry	N= 422			N= 858		
Baseline BMI	-0.12	-0.27	0.03	-0.17	-0.30	-0.05
BMI change	1.26e-03	-0.01	0.01	-0.08	-0.17	0.01
Murcia Twin register	N= 467			N= 831		
Baseline BMI	-0.09	-0.17	-0.02	-0.16	-0.23	-0.10
BMI change	-0.10	-0.20	8.59e-04	-0.12	-0.20	-0.01
Netherlands Twin Register	N= 1,418			N= 2,542		
Baseline BMI	-0.01	-0.05	0.04	-0.02	-0.06	0.04
BMI change	-2.19e-03	-4.54e-03	1.53e-04	-1.07e-03	-3.10e-03	9.51e-03
Swedish Twin Register	N= 3,704			N= 4,550		
Baseline BMI	-0.11	-0.13	-0.08	-0.13	-0.16	-0.10
BMI change	-2.04e-03	-7.80e-03	0.01	-0.02	-0.03	-0.01
North America						
Colorado Twin Registry	N= 649				N= 829	
Baseline BMI	0.11	-0.12	0.34	-0.10	-0.21	1.55e-03
BMI change	-0.01	-0.07	0.08	-0.02	-0.03	-0.01
NAS-NRC Twin Registry	N= 8,692			N= 0		
Baseline BMI	-0.01	-0.02	0.01	-	-	-
BMI change	-0.01	-0.02	-6.57e-03	-	-	-
Vietnam Era Twin Study of Aging	N= 743			N= 0		
Baseline BMI	-0.11	-0.24	0.01	-	-	-
BMI change	-0.04	-0.10	0.07	-	-	-
Washington State Twin Registry	N= 1,994			N= 3,883		
Baseline BMI	-0.13	-0.20	-0.05	-0.31	-0.38	-0.24
BMI change	-0.01	-0.08	0.05	-0.03	-0.10	0.03
Asia						
Korea Twin-Family Register	N= 77			N= 123		
Baseline BMI	0.02	-0.05	0.10	-0.09	-0.16	-0.02
BMI change	0.01	-0.01	0.02	-0.04	-0.12	0.03
Australia						
Queensland Twin Register	N= 3,394			N= 3,472		
Baseline BMI	-0.11	-0.16	-0.05	-0.18	-0.22	-0.14
BMI change	-0.06	-0.10	-0.03	-0.05	-0.08	-0.01
Crochan Q test	Cochran's Q		p-value	Cochran's Q		p-value
Baseline BMI	120.97		3.95e-20	125.38		4.06e-22
BMI change	42.88		2.35e-05	65.00		4.04e-10

Caption: For the study of the associations, LME models were used. The models are: i) Baseline BMI~ Maximum Educational attainment + Zygosity + Baseline Age + (1|Family ID) and ii) BMI change~ Maximum Educational attainment + Zygosity + Baseline Age +

Baseline BMI + (1|Family ID). The estimates are displayed along with the 95% confidence interval. Cochran's Q tests were performed to assess heterogeneity of the associations across cohorts. **Abbreviations:** BMI: Body mass index; CI: confidence interval; LL: lower limit; UL: upper limit; NAS-NRC: National Academy of Sciences-National Research Council.

Supplementary Table 3. Sensitivity analysis for the associations of educational attainment (in standardized education years) with BMI baseline (kg/m^2) and BMI change (kg/m^2 per decade) in pooled data by sex.

	Men (N=7,360)			Women (N=7,642)		
	**β**	**95%CI**		**β**	**95%CI**	
		**LL**	**UL**		**LL**	**UL**
Baseline BMI	-0.06	-0.07	-0.05	-0.13	-0.15	-0.11
BMI change	-0.01	-0.02	-4.25e-03	-0.01	-0.02	-1.12e-03

Caption: Associations for the sensitivity analysis including smoking behaviour as a covariate were carried out using LME models. The models are: i) BMI baseline~ Maximum Educational attainment + cohort ID + baseline smoking status + Zygosity + Baseline Age + (1|Family ID) and ii) BMI change~ Maximum Educational attainment + cohort ID + baseline smoking status + Zygosity + Baseline Age + BMI baseline + (1|Family ID). The estimates are displayed along with the 95% confidence interval. **Abbreviations:** BMI: Body mass index; CI: confidence interval; LL: lower limit; UL: upper limi

Supplementary Table 4. Sensitivity analysis for the associations of educational attainment (in standardized education years) with BMI baseline (kg/m^2) and BMI change (kg/m^2 per decade) at different stages of the adulthood in pooled data by sex.

	**Men**			**Women**		
	**β**	**95%CI**		**β**	**95%CI**	
		**LL**	**UL**		**LL**	**UL**
early midlife adulthood (30-50 years old)	N = 9,049			N = 10,402		
Baseline BMI	-0.09	-0.11	-0.07	-0.12	-0.15	-0.10
BMI change	-5.69e-03	-0.01	4.44e-03	-0.02	-0.03	-8.45e-03
midlife adulthood (51-65 years old)	N = 1,093			N = 1,799		
Baseline BMI	-0.04	-0.11	0.01	-0.09	-0.17	-0.02
BMI change	-0.04	-0.10	0.01	-0.10	-0.17	-0.03
Older adulthood (>66 years old)	N = 171			N = 374		
Baseline BMI	-0.12	-0.27	0.03	-0.24	-0.41	-0.08
BMI change	-0.11	-0.25	0.02	-0.02	-0.17	0.13

Caption: Sensitivity analyses dividing adulthood into different stages were performed using linear mixed-effects (LME) models, specified identically to the main association analyses. Outcomes were baseline BMI or BMI change, with educational attainment as the primary covariate. Models were adjusted for zygosity, cohort ID and baseline age; baseline BMI was additionally included as a covariate in the model of BMI change. The estimates are displayed along with the 95% confidence interval. **Abbreviations:** BMI: Body mass index; CI: confidence interval; LL: lower limit; UL: upper limit.

Supplementary Table 5. Intraclass correlations for baseline body mass index (BMI), its changes (kg/m^2 per ten years) and standardized education years in pooled data by sex and zygosity.

Variables of study	Men						Women						Opposite sex dizygotic (N=1,729)		
	MZ (N= 4,818)			DZ (N=6,214)			MZ (N=4,044)			DZ (N=5,324)					
	Estimate	95% CI		Estimate	95% CI		Estimate	95% CI		Estimate	95% CI		Estimate	95% CI	
		LL	UL		LL	UL		LL	UL		LL	UL		LL	UL
BMI baseline	0.78	0.77	0.79	0.57	0.55	0.59	0.77	0.76	0.78	0.55	0.53	0.57	0.37	0.32	0.41
BMI change	0.11	0.08	0.14	0.08	0.07	0.09	0.19	0.16	0.22	0.13	0.11	0.15	0.11	0.06	0.15
Standardized education years	0.70	0.69	0.71	0.52	0.50	0.54	0.72	0.71	0.74	0.48	0.46	0.50	0.36	0.32	0.40

Caption: Intraclass correlations for baseline BMI, BMI changes and standardized education years in pooled data from Europe, Asia, Australia, and North America are summarized by sex and zygosity with correlation coefficients besides their 95% confidence interval. All the correlations are highly significant (all $p<0.005$). Although only pooled ICCs are shown, MZ correlations were consistently less than twice DZ correlations supporting low heterogeneity and justifying pooling. **Abbreviations:** MZ: Monozygotic; DZ: Dizygotic; CI: Confidence interval; LL: Lower limit; UL: Upper limit.

Supplementary Table 6. Model fit statistics of baseline body mass index (BMI), its changes (kg/m^2 per decade) and educational attainment in the pooled data comparing different genetic models.

Variables of study	Full ACE model (reference model)		ACE model without sex-specific genetic effect (1)		ACE model with same parameter estimates for men and women (2)		Full AE model (3)		AE model without sex-specific genetic effect (4)		AE model with same parameter estimates for men and women (3)	
	-2LL	d.f	Δ -2 LL	p value	Δ -2 LL	p value	Δ -2 LL	p value	Δ -2 LL	p value	Δ -2 LL	p value
Baseline BMI	21186.33	4225	0.49	0.48	370.05	6.78e-48	0.38	0.53	62.46	2.71e-15	73.01	1.39e-16
BMI change	16800.42	4225	0.61	0.42	200.34	5.48e-26	0.29	0.37	100.24	8.08e-17	207.44	6.31e-29
EA	21010.21	4225	7.65	5.94e-03	89.59	2.68e-19	31.29	8.25e-12	53.75	2.14e-12	18.22	1.96e-05

Caption: Full ACE model is compared against the rest of the models displaying the -2 log likelihood and degrees of freedom for the reference model and the differences in -2log likelihood and the p value in the models compared with the reference one. **Abbreviations:** -2LL (-2 log-likelihood); d.f. (degrees of freedom); Δ (change); ACE (additive genetic/ shared environment/ unique environment) model; AE (additive genetic/ unique environment) model. (1) Compared to full ACE model (Δ d.f. 1); (2) Compared to the ACE model without sex-specific genetic effect (Δ d.f. 1); (3) Compared to the full ACE model (Δ d.f. 2); (4) Compared to the full AE model (Δ d.f. 1); (5) Compared to the AE model without sex-specific genetic effect (Δ d.f. 1)

Supplementary Table 7. Contribution of additive genetic and unique environmental factors to the previously identified associations between EA and BMI trajectory components (Baseline BMI and BMI change) at cohort specific level analysis, by sex.

Variables of study	Men						Women					
	Additive genetic correlation			Unique environmental correlations			Additive genetic correlation			Unique environmental correlation		
	r_A	95% CI		r_E	95% CI		r_A	95% CI		r_E	95% CI	
		LL	UL		LL	UL						
Europe												
Finnish old cohort												
Baseline BMI	-0.16	-0.19	-0.13	-0.04	-0.10	-0.01	-0.20	-0.22	-0.18	0.03	-0.02	0.08
BMI change	-	-	-	-	-	-	-	-	-	-	-	-
Italian Twin Registry												
Baseline BMI	-	-	-	-	-	-	-0.90	-1.00	-0.78	-0.24	-0.52	0.05
BMI change	-	-	-	-	-	-	-	-	-	-	-	-
Murcia Twin register												
Baseline BMI	-0.31	-0.49	-0.14	0.17	-0.03	0.37	-0.43	-0.58	-0.28	0.01	-0.11	0.13
BMI change	0.08	-0.87	0.87	-0.01	-0.23	0.20	-0.12	-0.44	0.10	-0.01	-0.13	0.10
Netherlands Twin Register												
Baseline BMI	0.31	0.24	0.38	0.15	0.01	0.29	0.32	0.26	0.39	0.13	-0.01	0.27
BMI change	-	-	-	-	-	-	-	-	-	-	-	-
Swedish Twin Register												
Baseline BMI	-0.25	-0.29	-0.21	0.03	-0.02	0.08	-0.25	-0.28	-0.22	0.03	-0.01	0.07
BMI change	-	-	-	-	-	-	0.01	-0.04	0.05	-0.01	-0.05	0.03
North America												
Colorado Twin Register												
BMI baseline	-	-	-	-	-	-	-	-	-	-	-	-
BMI change	-	-	-	-	-	-	-	-	-	-	-	-
NAS-NRC Twin Registry												
BMI baseline	-	-	-	-	-	-	-	-	-	-	-	-
BMI change	-0.16	-0.21	-0.12	0.01	-0.02	0.05	-	-	-	-	-	-
Vietnam Era Twin Study of Aging												
BMI baseline	-	-	-	-	-	-	-	-	-	-	-	-
BMI change	-	-	-	-	-	-	-	-	-	-	-	-
Washington State Twin Registry												
Baseline BMI	-0.09	-0.20	0.02	-0.13	-0.20	-0.03	-0.04	-0.15	0.09	-0.16	-0.23	-0.05
BMI change	-	-	-	-	-	-	-	-	-	-	-	-
Asia												
Korea Twin-Family Register												
Baseline BMI	-	-	-	-	-	-	-0.12	-0.30	0.07	-0.27	-0.45	-0.02

BMI change	-	-	-	-	-	-	-	-	-	-	-	-
Australia												
Queensland Twin Register												
Baseline BMI	-0.17	-0.28	-0.05	-0.01	-0.09	0.08	-0.12	-0.21	-0.04	-0.10	-0.15	-0.04
BMI change	-0.19	-0.31	-0.07	-0.02	-0.11	0.06	-0.24	-0.39	-0.12	0.01	-0.04	0.06
Crochan Q test	Cochran's Q		p-value	Cochran's Q		p-value	Cochran's Q		p-value	Cochran's Q		p-value
Baseline BMI	195.14		2.05e-39	21.27		3.38e-03	441.93		1.52e-89	46.10		1.37e-06
BMI change	1.76		0.62	14.72		2.06e-03	12.59		5.58e-03	0.49		0.92

Caption: Additive genetic (r_A) and unique environmental (r_E) correlations of EA with Baseline BMI and BMI change were obtained for the previously found significant associations at cohort specific level using the genetic twin models, which decompose phenotypic covariation into genetic and environmental effects. Cochran's Q tests were performed to assess heterogeneity of these correlations across cohorts and sex.
Abbreviations: r_A: Additive genetic correlation coefficient; r_E; Specific environmental correlation coefficient; LL: Lower limit; UL: Upper limit
